# Supplementary material for: Deficiency of GABARAP but Not Its Paralogs Causes Enhanced EGF-Induced EGFR Degradation
Source: Cells. 2020 May 22;9(5):1296. doi: 10.3390/cells9051296 (PMC7291022; doi:10.3390/cells9051296)
Supplement: Supplementary file 1 [file cells-09-01296-s001.zip › final SM 6.4/cells-745021 final Supplemenatary materials.pdf]

Supplemental Figures

Deficiency of GABARAP but not its paralogs causes enhanced EGF-induced EGFR degradation

Jochen Dobner[1], Indra M. Simons[1, 2], Kerstin Rufinatscha[3], Sebastian Hänsch [4], Melanie Schwarten[2], Oliver H. Weiergräber[2], Iman Abdollahzadeh[2, 5], Thomas Gensch[5], Johannes G. Bode[3], Silke Hoffmann[2], Dieter Willbold[1, 2] *

[1] Institut für Physikalische Biologie, Heinrich-Heine-Universität Düsseldorf, Universitätsstraße 1, 40225 Düsseldorf, Germany
[2] Institute of Biological Information Processing: Structural Biochemistry (IBI-7), Forschungszentrum Jülich, 52425 Jülich, Germany
[3] Department of Gastroenterology, Hepatology and Infectiology, University Hospital, Heinrich-Heine-Universität Düsseldorf, Moorenstraße 5, 40225 Düsseldorf, Germany
[4] Department of Biology, Center for Advanced Imaging (CAi), Heinrich-Heine-Universität Düsseldorf, Universitätsstraße 1, 40225 Düsseldorf, Germany
[5] Institute of Biological Information Processing – Molecular and Cell Physiology (IBI-1), Forschungszentrum Jülich, 52425 Jülich, Germany

Supplementary Figure S1 is related to Figures 1-8
Supplementary Figure S2 is related to Figure 1
Supplementary Figure S3 is related to Figure 5
Supplementary Figure S4 is related to Figure 7 and Movie S1
Supplementary Figure S5 is related to Figure 7 and Movie S2
Supplementary Figure S6 is related to Figures 1-4, 6, 8 and Figure S2
Supplementary Table S1 is related to Figures 1-8
Movie S1 is related to Figures 7 and S4
Movie S2 is related to Figures 7 and S5

Supplemental Figures S1 - 6, Table S1, Movies S1 - 2

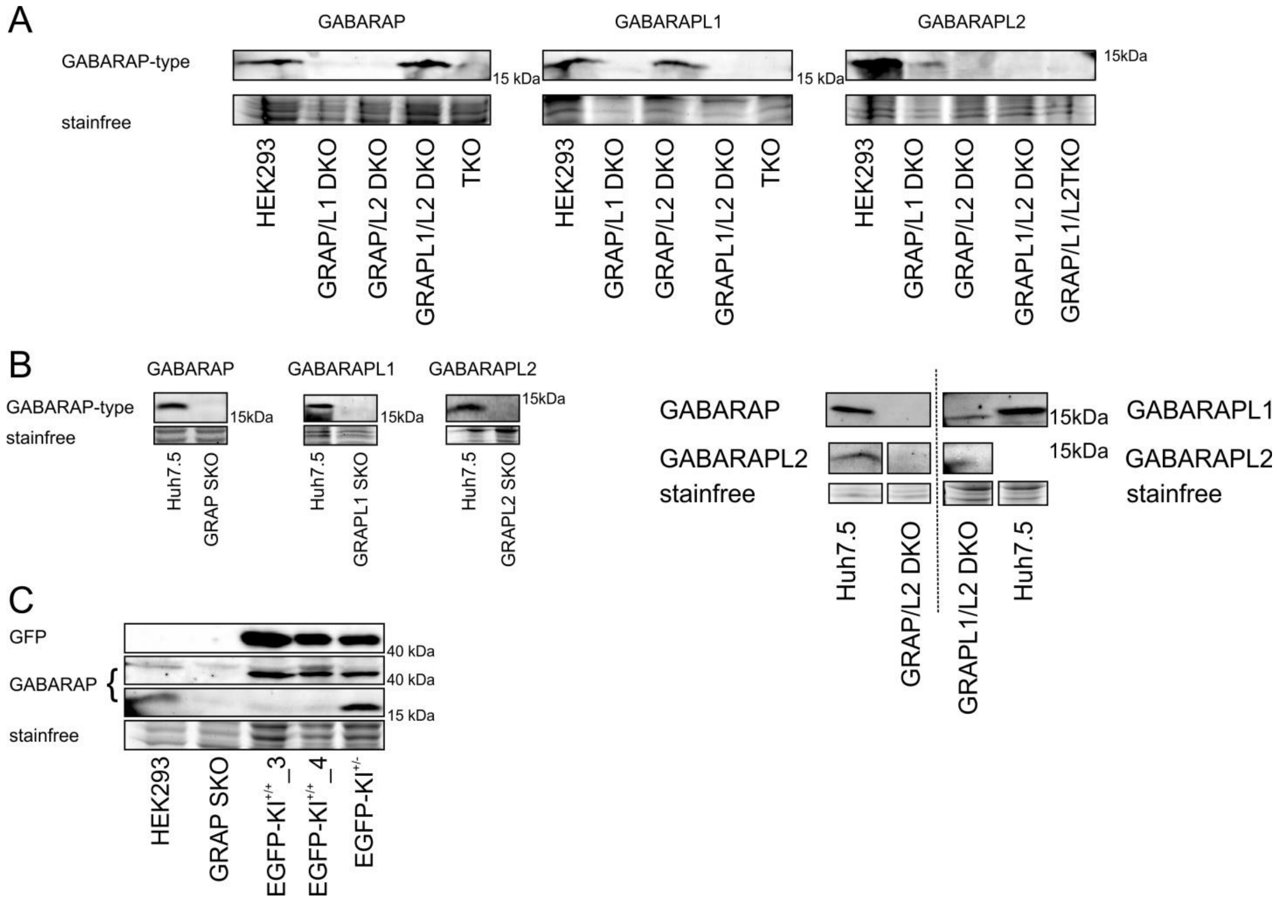

Figure S1: Verification of CRISPR/Cas9 knockout and knock-in cell lines on protein level.
Whole cell protein lysates were isolated and analyzed for presence of the indicated GABARAP subfamily member. (A) HEK293 based clonal KO cell lines. (B) Huh7.5 based clonal KO cell lines. (C) HEK293 based KI clonal lines. GRAP = GABARAP, GRAPL1 = GABARAPL1, GRAPL2 = GABARAPL2, TKO = GABARAP/L1/L2 TKO. SKO = single knockout, DKO = double knockout, TKO = triple knockout. Figure S7 G shows uncropped source blots

Table S1: CRISPR sequence details and genotyping results of the knockout cell lines used.

HEK293 GABARAP, GABARAPL1 and GABARAPL2 SKOs have already been published (Simons *et al.*, 2019).

Gene Symbol	Uniprot	GeneID/ Location	Targeting strategy	CRISPR gRNA (PAM)	Main clone	Unique Alleles	Mutation	Protein Impact
**Huh7.5**								
GABARAP	O95166	11337/NC_000017.11	first exon	GGATCTTCTCGCCCTCAGAG CGG	3	1	c.[152_153insT]	p.[fs*0]
GABARAPL1	Q9H0R8	23710/NC_000012.12	second exon	AGAGAAGGCTCCAAAAGCC AGGG	G5	3	c.[352_358_del];[353_356del];[154_355del]	p.[K38Gfs*9];[K38Gfs*11];[K38Sfs*3]
GABARAPL2	P60520	11345/NC_000016.1	second exon	TCCCACAGAACACAGATGCG TGG	F6	1	c.[179_180insT]	p.[C15Lfs*27]
GABARAP/L2 DKO	O95166	11337/NC_000017.11	first exon	GGATCTTCTCGCCCTCAGAG CGG	G8	2	c.[152_153insTT];[152_154insGG]	p.[E17Lfs*36];[E17Gfs*14]
	P60520	11345/NC_000016.1	second exon	TCCCACAGAACACAGATGCG TGG		2	c.[179_180insT];[179_180insTG]	p.[C15Lfs*27];[V16Afs*15]
GABARAPL1/L2 DKO	Q9H0R8	23710/NC_000012.12	second exon	AGAGAAGGCTCCAAAAGCC AGGG	F5	2	c.[354_355del];[355del]	p.[K38Nfs*3];[A39Pfs*10]
	P60520	11345/NC_000016.1	second exon	TCCCACAGAACACAGATGCG TGG		1	c.[179_180insT]	p.[C15Lfs*27]
**HEK293**								
GABARAP	O95166	11337/NC_000017.11	first exon	GGATCTTCTCGCCCTCAGAG CGG	C2	1	c.[152_153insT]	p.[fs*0]
GABARAPL1	Q9H0R8	23710/NC_000012.12	second exon	AGAGAAGGCTCCAAAAGCC AGGG	C10	2	c.[352_357del];[353_356]	p.[K38Tfs*10];[K38Nfs*3]

GABARAPL2	P60520	11345/NC_000016.1	second exon	TCCCACAGAACACAGATGCG TGG	#8	1	c.[179_180insT]	p.[C15Lfs*27]
GABARAP/L1 DKO	O95166	11337/NC_000017.11	first exon	GGATCTTCTCGCCCTCAGAG CGG	A11 (based on C10)	2	c.[152_153insT];[148_151del]	p.[fs*0];[K23Nfs*6]
GABARAP/L2 DKO	P60520	11345/NC_000016.1	second exon	TCCCACAGAACACAGATGCG TGG	#8 (based on C2)	1	c.[179_180insT]	p.[C15Lfs*27]
GABARAPL1/L2 DKO	P60520	11345/NC_000016.1	second exon	TCCCACAGAACACAGATGCG TGG	B3 (based on C10)	1	c.[179_180insT]	p.[C15Lfs*27]
GABARAP/L1/L2 TKO	O95166<br>P60520	11337/NC_000017.11<br>11345/NC_000016.1	first exon<br>second exon	GGATCTTCTCGCCCTCAGAG CGG<br>TCCCACAGAACACAGATGCG TGG	#3 (based on C10)	1<br>1	c.[152_153insT]<br>c.[179_180insT]	p.[fs*0]<br>p.[C15Lfs*27]
GABARAP	O95166	11337/NC_000017.11	first exon +linearized HDR plasmid	TACACGAACTTCATCCTCCC GGG	3	1	c.[ins717+21bpEGFP+linker]	p.[ins239+7aaEGFP+linker]

Formatting of indels detected in the knockout cell lines (Mutation column) and their resulting proteins (Protein impact column) is according to Human Genome Variation Society (http://varnomen.hgvs.org/). Mutation positions are determined in respect to the canonical isoform annotated in Uniprot, if more than one form exists. The numbers after the asterisks represent the number of amino acids present from the first amino acid changed to the next sequential stop codon. del, deletion; ins, insertion; c., coding DNA; p., protein; fs, frame shift; *, stop codon.

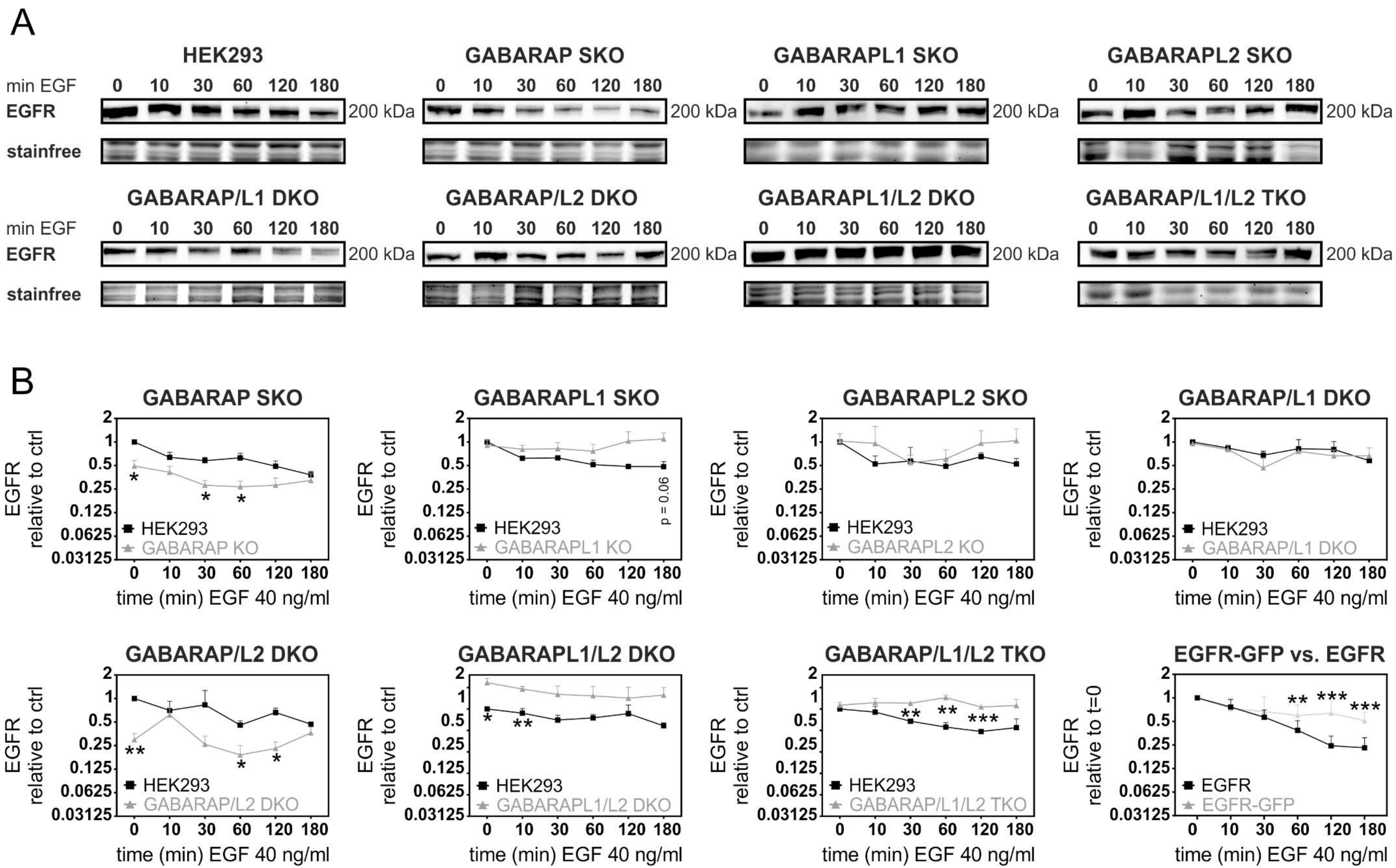
A
HEK293
GABARAP SKO
GABARAPL1 SKO
GABARAPL2 SKO
min EGF
0 10 30 60 120 180
EGFR
200 kDa
stainfree
GABARAP/L1 DKO
GABARAP/L2 DKO
GABARAPL1/L2 DKO
GABARAP/L1/L2 TKO
B
GABARAP SKO
GABARAPL1 SKO
GABARAPL2 SKO
GABARAP/L1 DKO
GABARAP/L2 DKO
GABARAPL1/L2 DKO
GABARAP/L1/L2 TKO
EGFR-GFP vs. EGFR
EGFR relative to ctrl
EGFR relative to t=0
2
1
0.5
0.25
0.125
0.0625
0.03125
time (min) EGF 40 ng/ml
HEK293
GABARAP KO
GABARAPL1 KO
GABARAPL2 KO
GABARAP/L1 DKO
GABARAP/L2 DKO
GABARAPL1/L2 DKO
GABARAP/L1/L2 TKO
EGFR
EGFR-GFP
p = 0.06

Figure S2: Total EGFR levels in response to EGF treatment in HEK293 KO cells transiently overexpressing EGFR-GFP.
(A) Cells were transfected with 2 µg EGFR-GFP expression plasmid and two days post transfection treated with 40 ng/ml EGF for the indicated time points. Afterwards, total EGFR protein levels in whole cell lysates were analyzed by immunoblot. Representative blots are shown for at least n = 3 independent experiments. (B) Densitometric analysis of at least n = 3 independent experiments. Controls are directly associated to each experiment. Kinetics of EGF-induced EGFR-GFP degradation in EGFR-GFP transfected HEK293 compared to endogenous EGFR degradation is shown (EGFR-GFP n = ≥ 23, EGFR n = 20). Quantification of EGFR(-GFP) protein levels was performed by normalization on stain-free loading control and calculated as percentage of HEK293 control cells at unstimulated conditions (t = 0). For comparison between EGFR-GFP overexpression and endogenous EGFR, kinetics of each level of EGFR expression (overexpression or endogenous) is displayed (t = 0 for each condition individually). Error bars represent standard error of means. Asterisks mark significant differences versus the corresponding time point of control cells and were calculated using independent t-test. $p \leq 0.05$ = *, $p \leq 0.01$ = **, $p \leq 0.001$ = ***. Respective wildtype controls were run on the same PAGE for each KO cell line and can be found in figure S7 H which also shows the uncropped source blots.

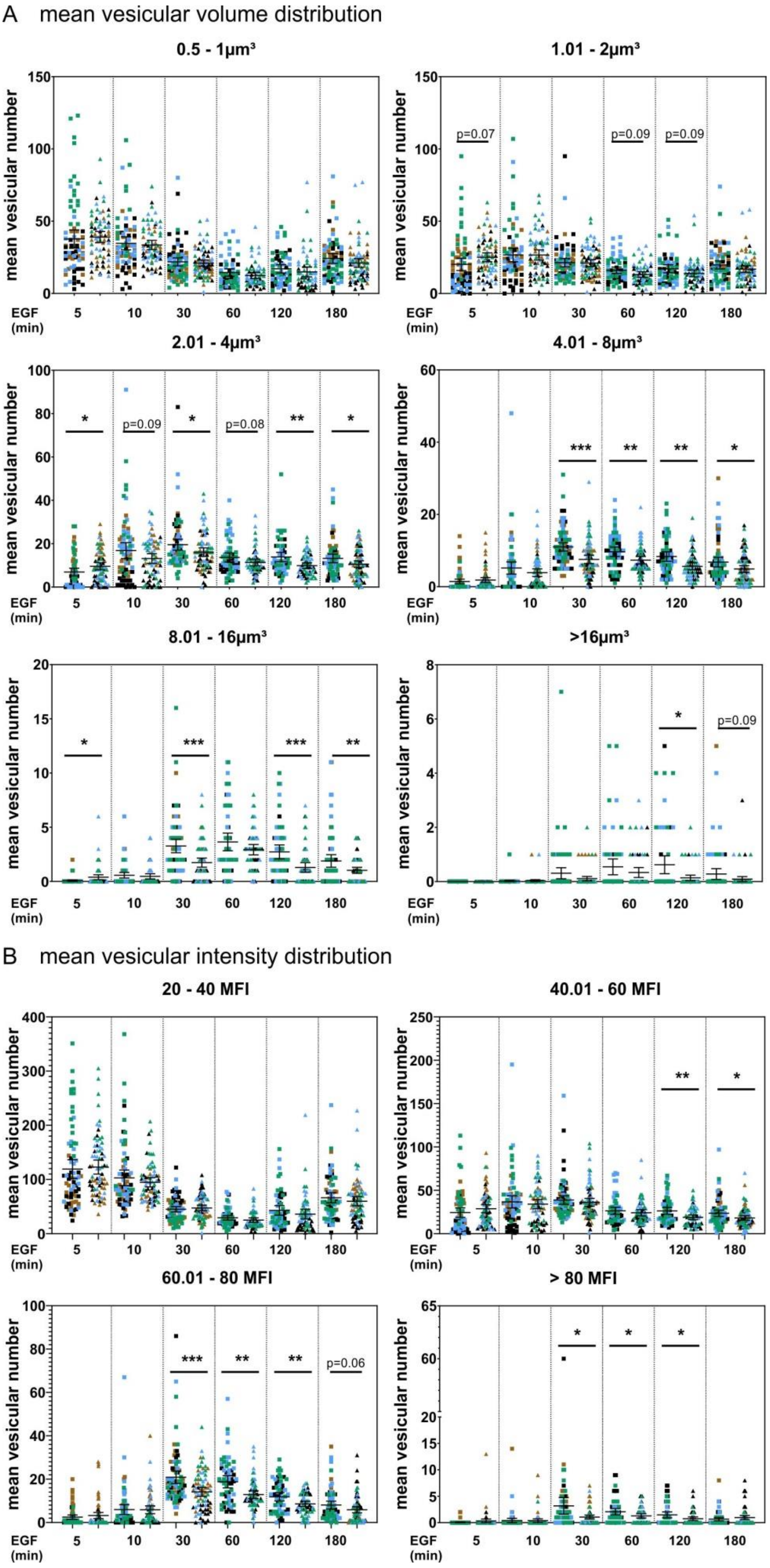
A mean vesicular volume distribution
0.5 - 1μm³
1.01 - 2μm³
p=0.07
p=0.09
p=0.09
2.01 - 4μm³
*
p=0.09
*
p=0.08
**
*
4.01 - 8μm³
***
**
**
*
8.01 - 16μm³
*
***
***
**
>16μm³
*
p=0.09
mean vesicular number
EGF (min)
5
10
30
60
120
180
B mean vesicular intensity distribution
20 - 40 MFI
40.01 - 60 MFI
**
*
60.01 - 80 MFI
***
**
**
p=0.06
> 80 MFI
*
*
*

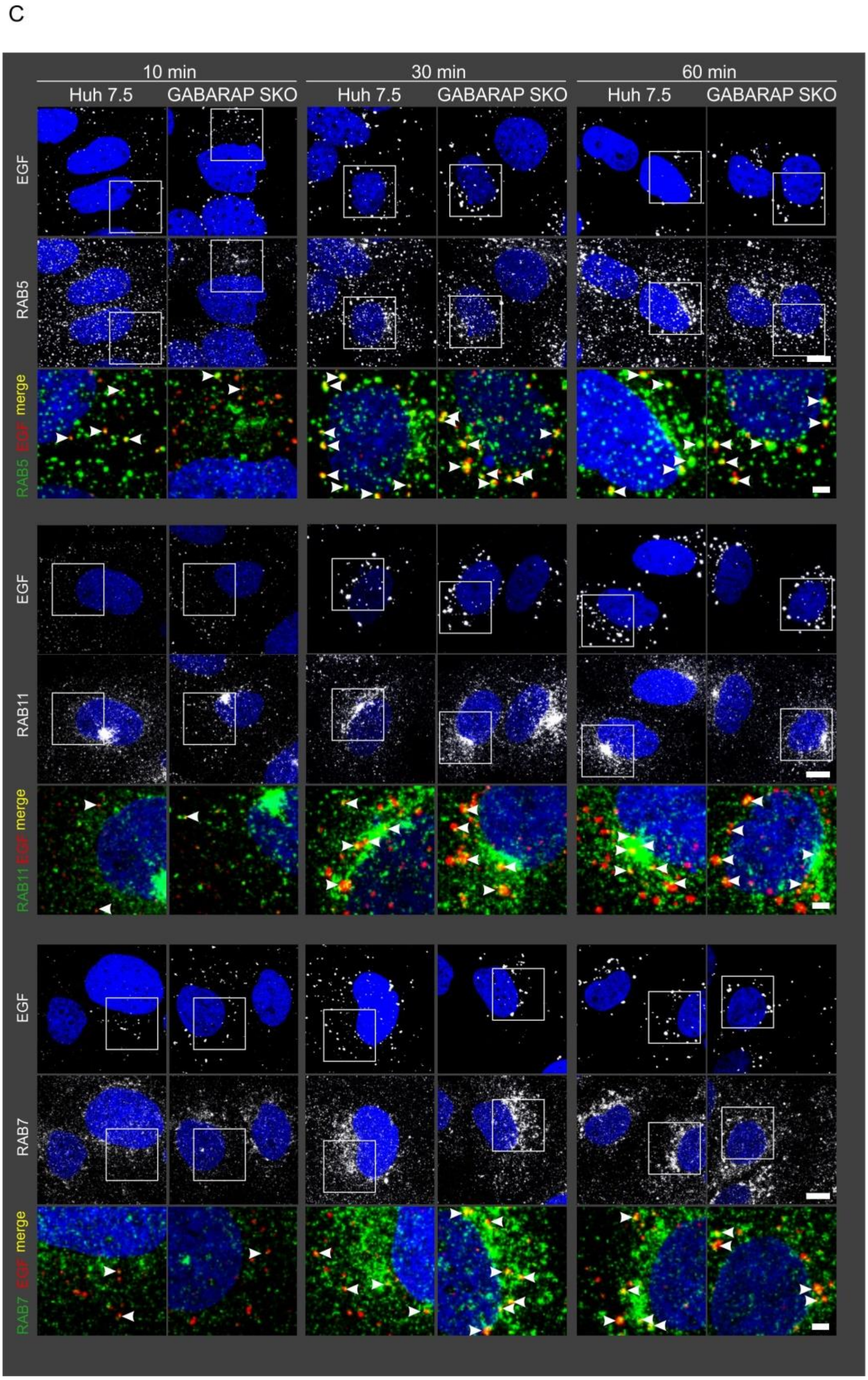

Figure S3: Analysis of EGF-Alexa647 containing vesicles of GABARAP SKO and Huh7.5 cells by Imaris and analysis of endosomal markers RAB 5, RAB11 and RAB7 in response to EGF treatment in GABARAP SKO and Huh7.5 control cells.

Vesicles of GABARAP SKO and Huh7.5 control cells which were treated with 40 ng/ml EGF-Alexa647 were modelled with Imaris imaging analysis software (described in detail in figure 5 and materials and methods section). (A) Mean vesicular volumes were largely unaffected by GABARAP-deficiency in GABARAP SKO cells although tendency for less vesicles with a diameter of $16 - 32\ \mu m^3$ was observable for GABARAP SKO cells after 30 min of treatment. Asterisks mark significant differences at indicated time points versus control cells as calculated using independent t-test. $p \leq 0.01$ = **. (B) Mean fluorescence intensities of EGF-Alexa647 positive vesicles classified into four arbitrary groups for GABARAP SKO and Huh7.5 control cells revealed significantly less vesicles with highest mean fluorescence intensities > 80 for GABARAP SKO cells compared to Huh7.5 control cells after 30 min of treatment. (B+C) Individual experiments are color-coded; > 50 cells per genotype and time point were analyzed. Error bars represent 95 % CI (C-E). Asterisks mark significant differences to the corresponding time point of control cells and were calculated using independent t-test. $p \leq 0.05$ = *, $p \leq 0.01$ = **, $p \leq 0.001$ = ***. (C) Huh7.5 and GABARAP SKO cells were pulse-treated with 40 ng/ml EGF-Alexa647 at 4 °C to allow binding to EGFR. After rigorous washing, cells were placed at 37 °C, fixed at distinct time points and stained for early (RAB5), recycling (RAB11) or late (RAB7) endosomes. All analyzed RAB proteins strongly accumulated within the first 30 min after EGF-Alexa647 pulse. Colocalization analysis of RAB proteins with EGF-Alexa647 (white arrowheads) revealed no alterations between GABARAP SKO and Huh7.5 control cells. MFI = mean fluorescence intensity Scale bar = 10 µm, scale bar zoom = 3 µm.

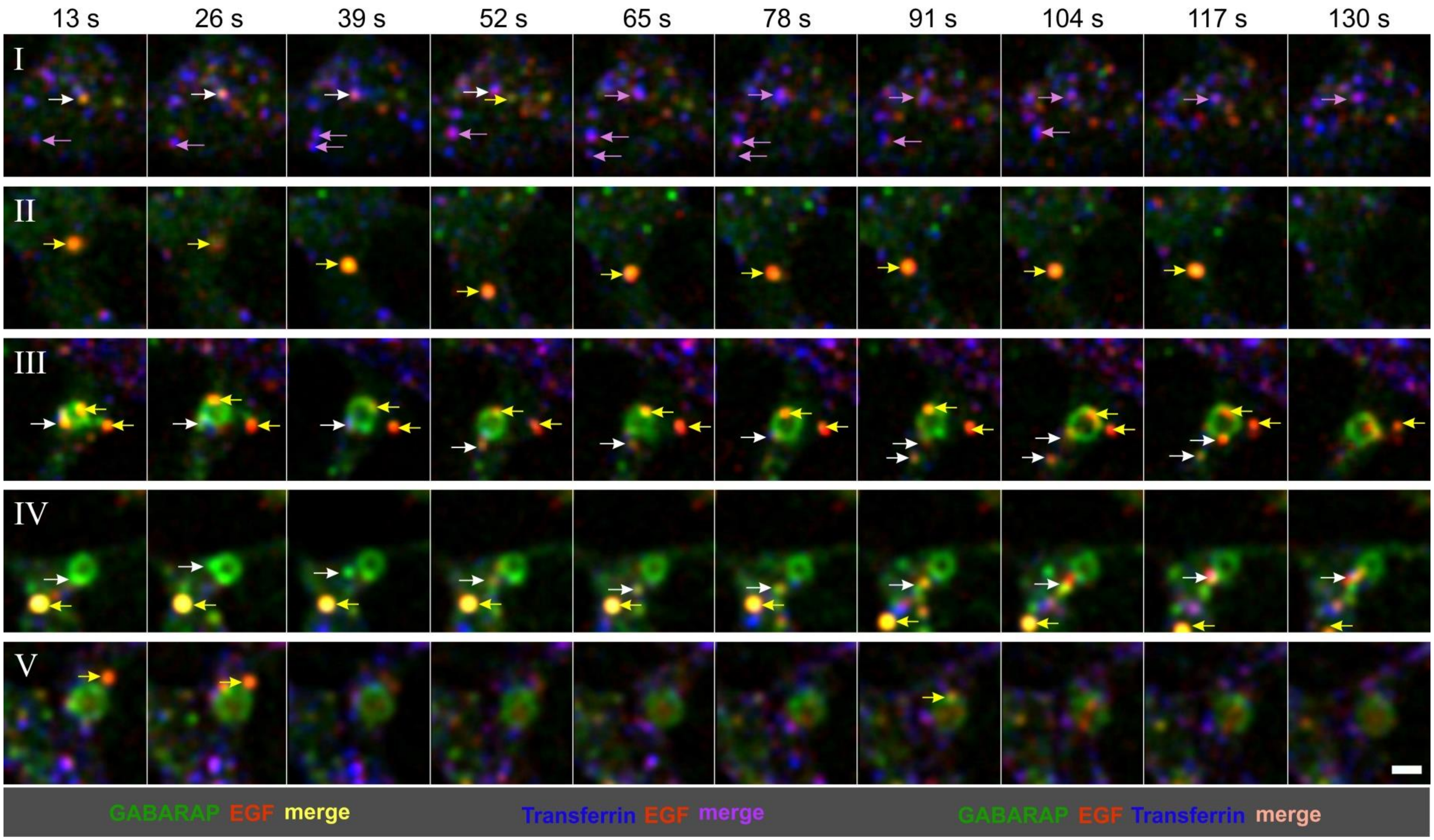

Figure S4: Montage of live cell imaging of HEK293 knock-in cells expressing GFP-GABARAP under the endogenous *GABARAP* promoter and stimulated with EGF-Alexa647 and Tf-Alexa555 by confocal laser scanning microscopy. HEK293 GFP-GABARAP KI cells were simultaneously treated with 40 ng/ml EGF-Alexa647 and 20 ng/ml Tf-Alexa555 for 60 min and imaged under live-cell conditions by laser scanning microscopy. The montage shows snapshots of regions of interest I to V indicated in figure 7 A, covering a 117 s time frame. Images were taken at intervals of 13 s. Selected GABARAP/EGF positive signals are marked with yellow arrows, GABARAP/EGF/Tf positive structures with white arrows and EGF/Tf positive vesicles with magenta arrows. Video can be found under movie S1. Scale bar = 3 µm.

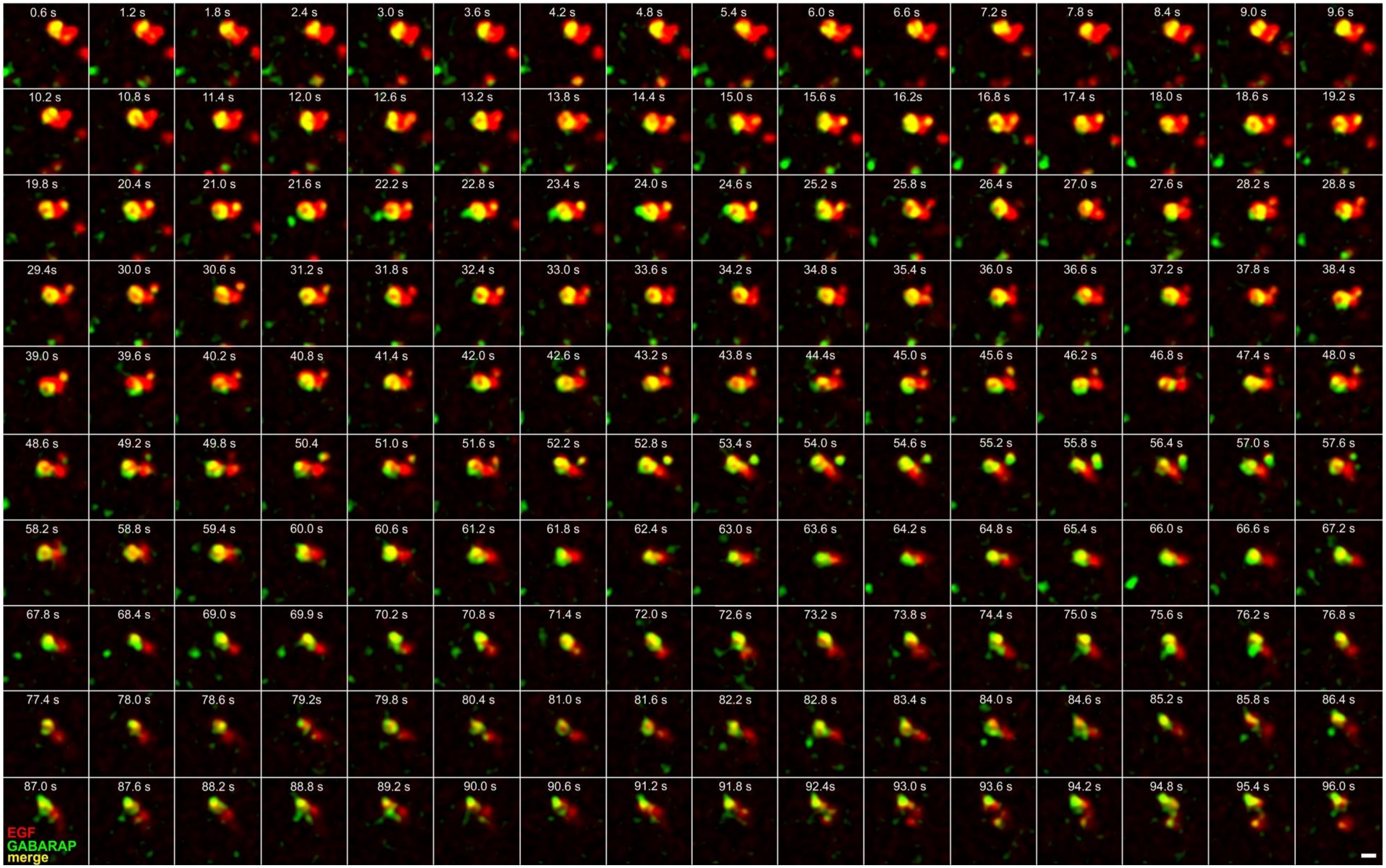

Figure S5: Live cell imaging of HEK293 KI cells expressing GFP-GABARAP under the endogenous *GABARAP* promoter and stimulated with EGF-Alexa647 by spinning disk confocal fluorescence microscopy.

HEK293 GFP-GABARAP KI cells were treated with 40 ng/ml EGF-Alexa647 for 60 min and imaged under live-cell conditions by spinning disk confocal fluorescence microscopy. The montage features selected time points for areas shown in figures 7 B to D. Dynamic vesicles which are GABARAP positive (green structures), EGF positive (red structures) or GABARAP/EGF double positive (yellow structures) are shown over a time course of 95.4 s with intervals of 0.6 s between images. GABARAP is shown in green and EGF in red. Video can be found under movie S2. Scale bar = 3μm.

A Source blots corresponding to figure 1

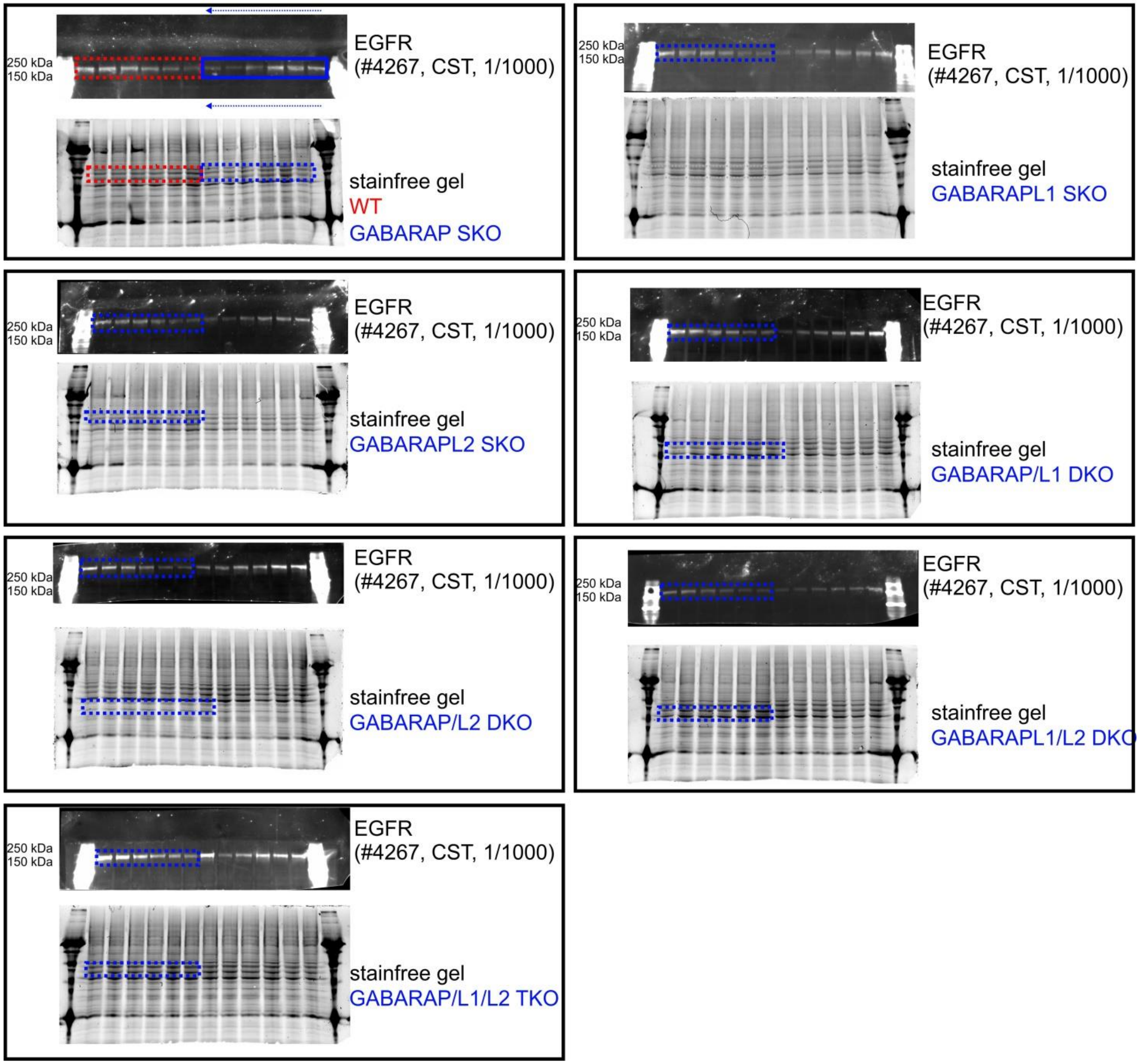

B Source blots corresponding to figure 2

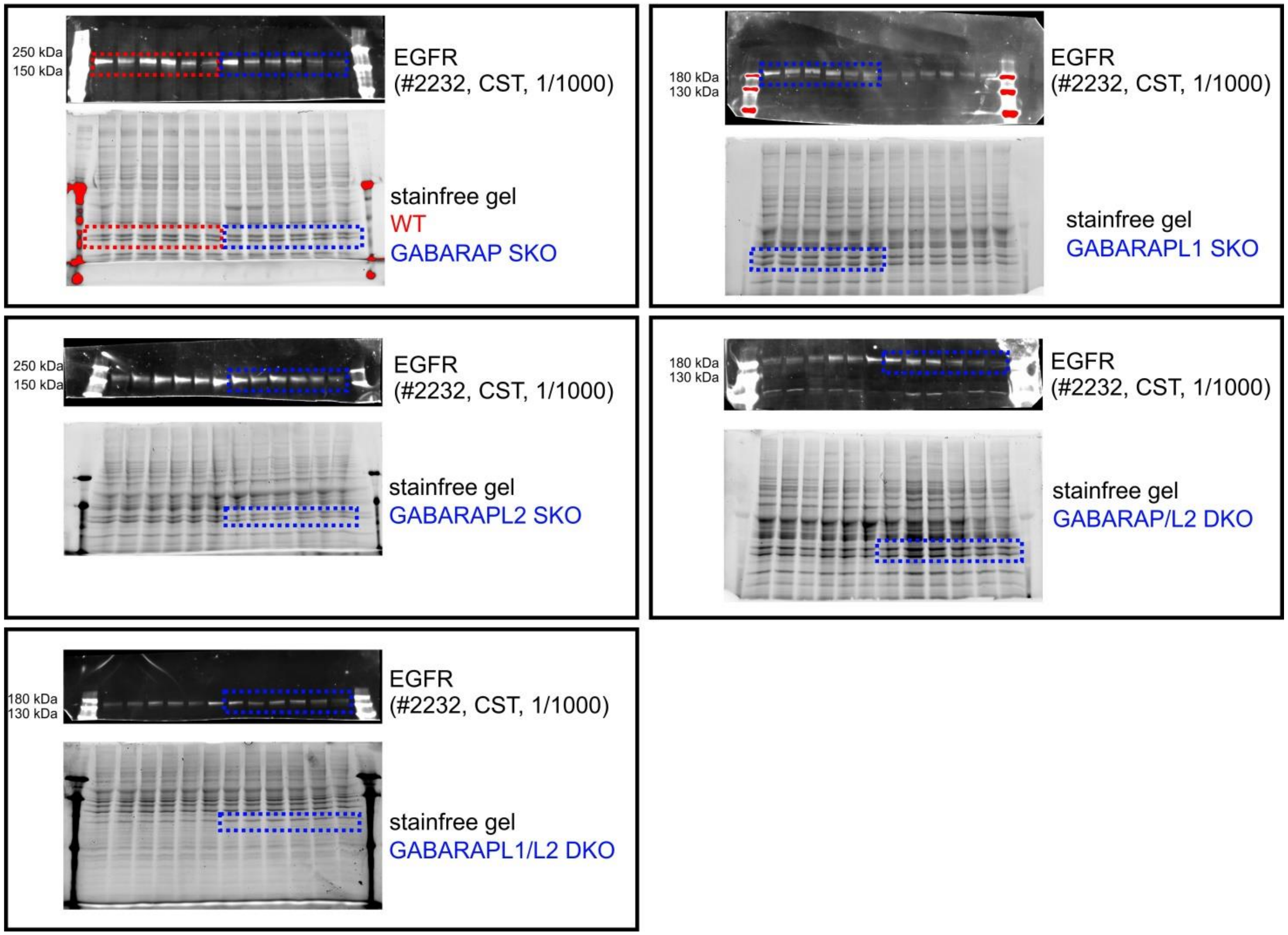

C Source blots corresponding to figure 3

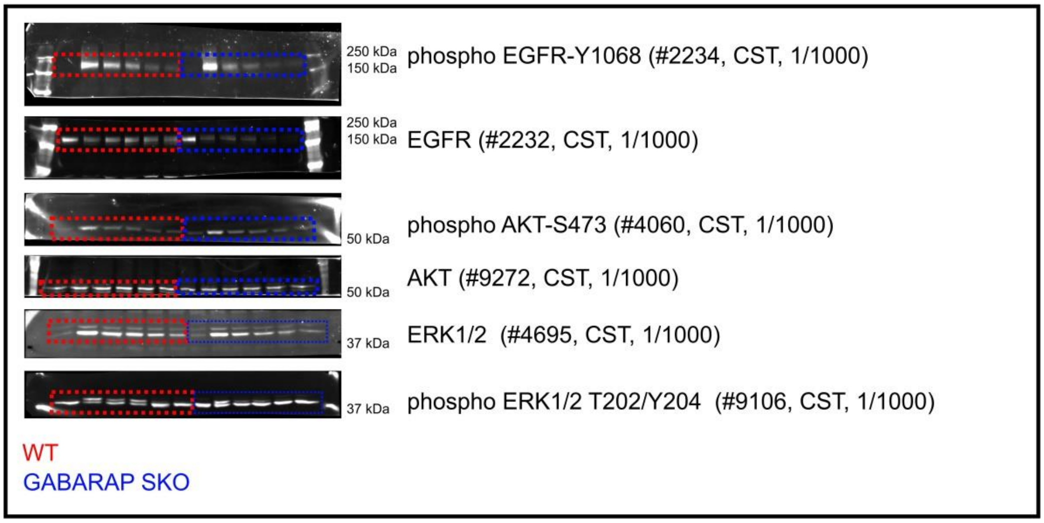

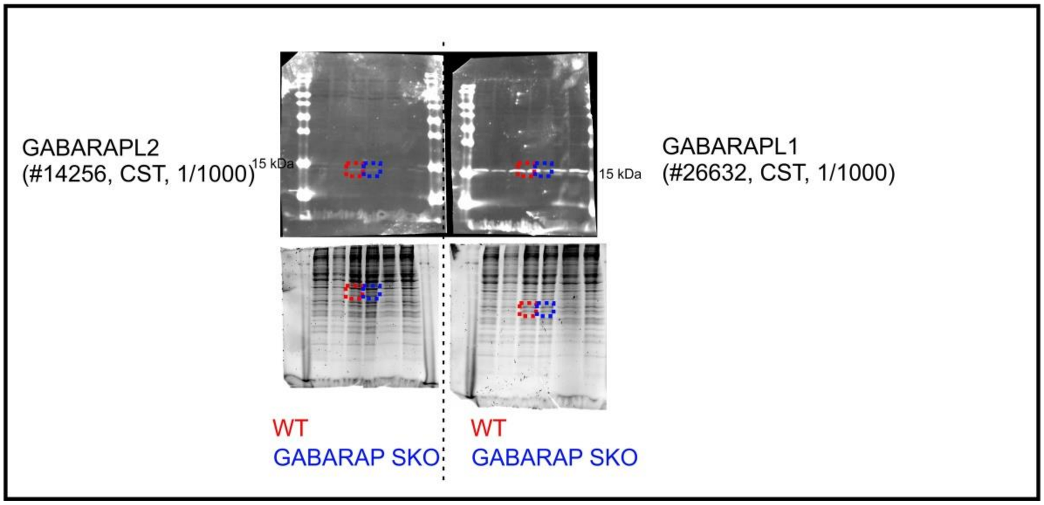

D Source blots corresponding to figure 4

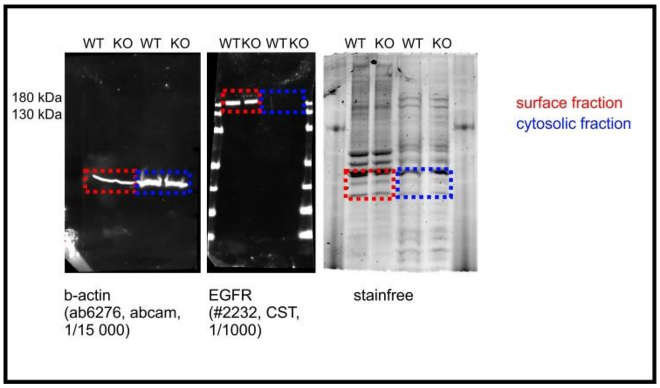

E Source blots corresponding to figure 6

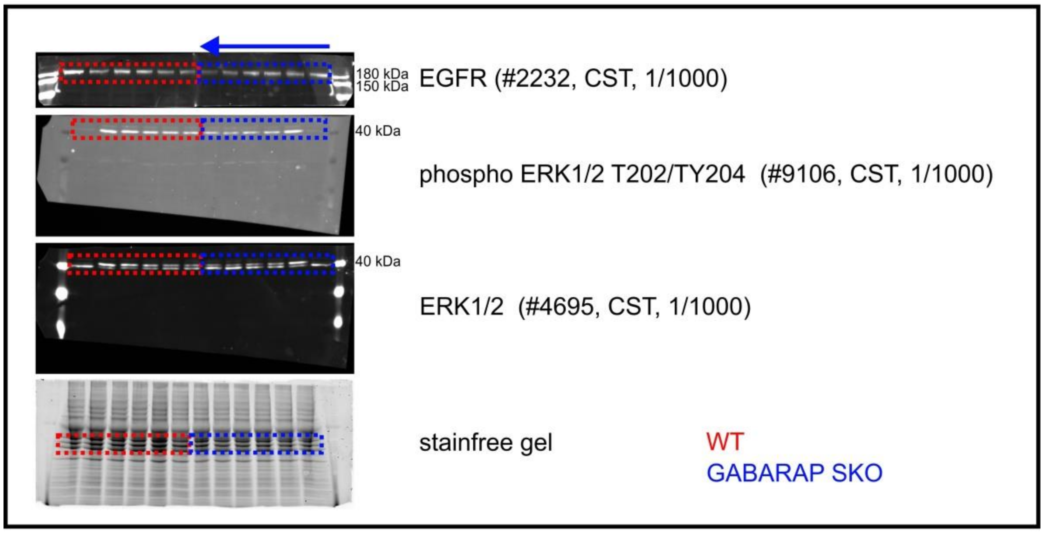

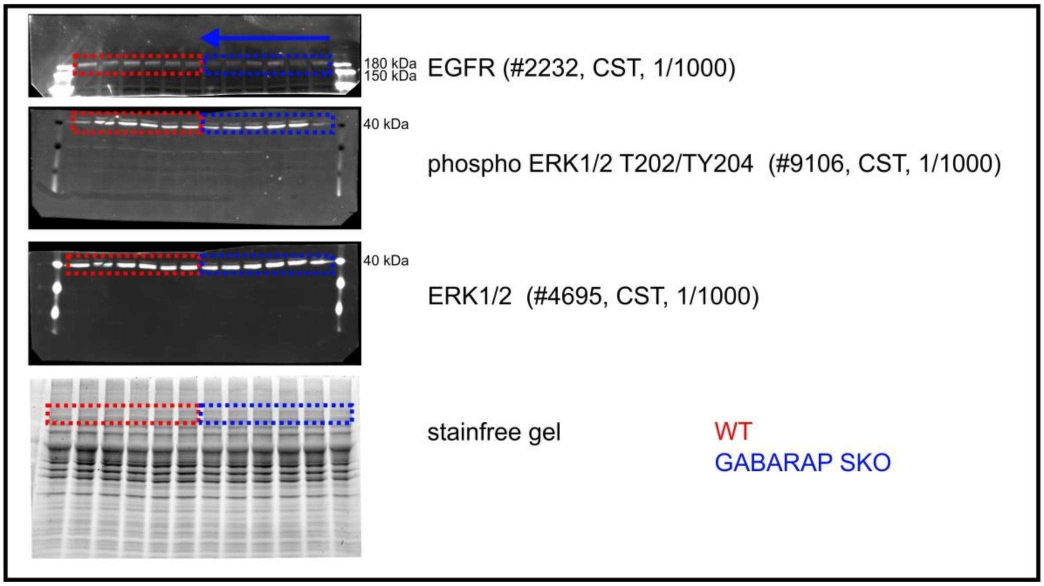

F Source blots corresponding to figure 8

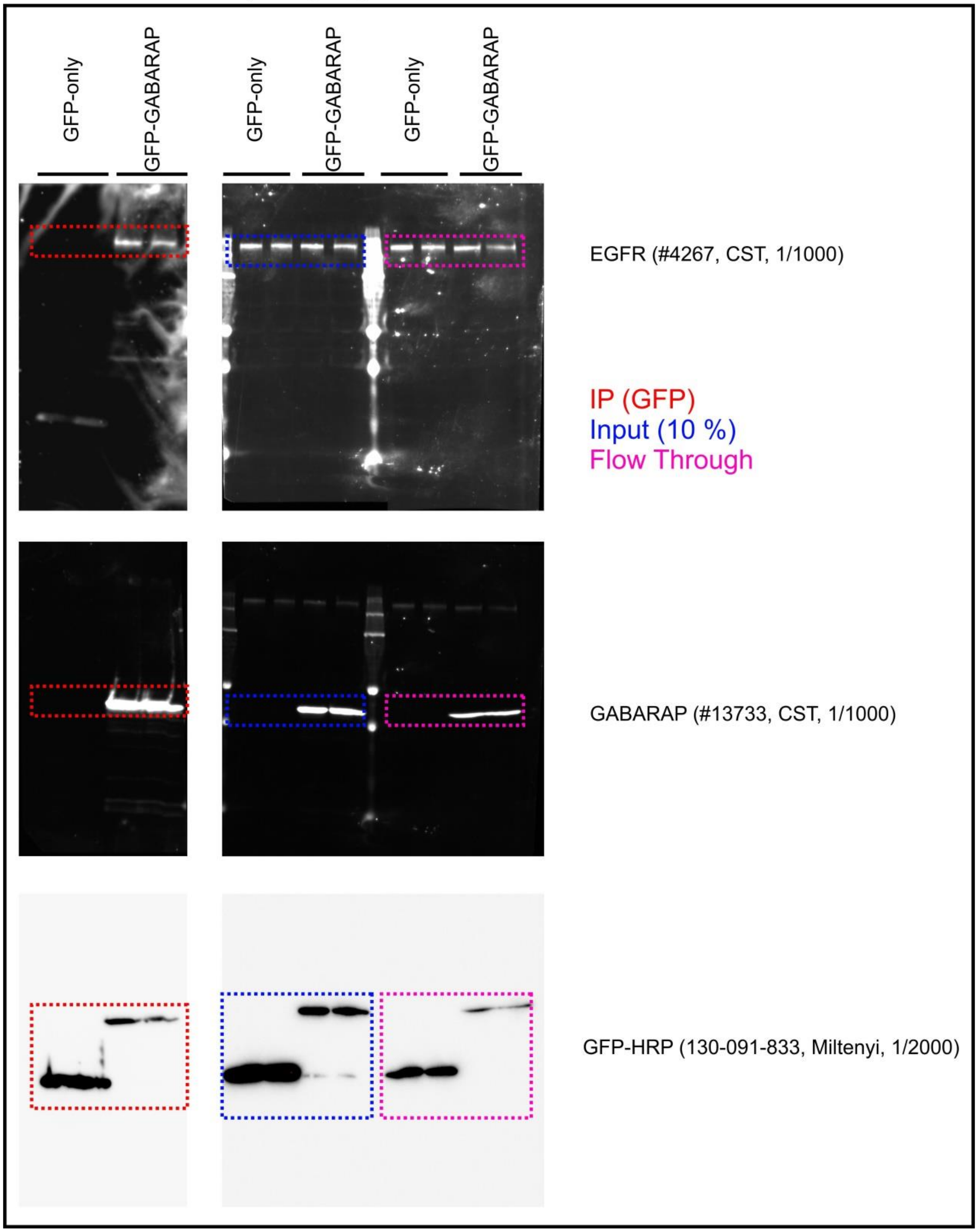

G Source blots corresponding to figure S1

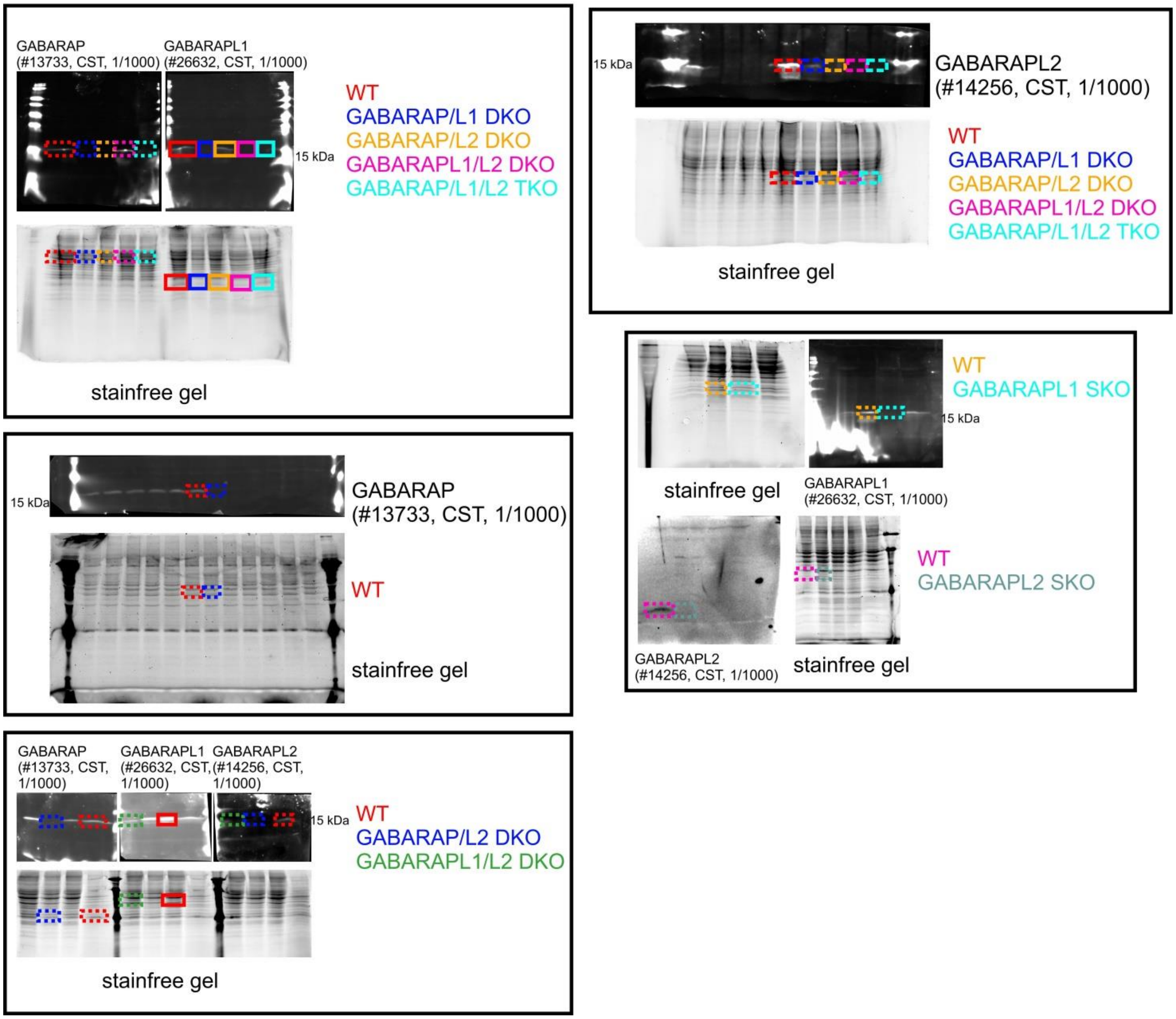

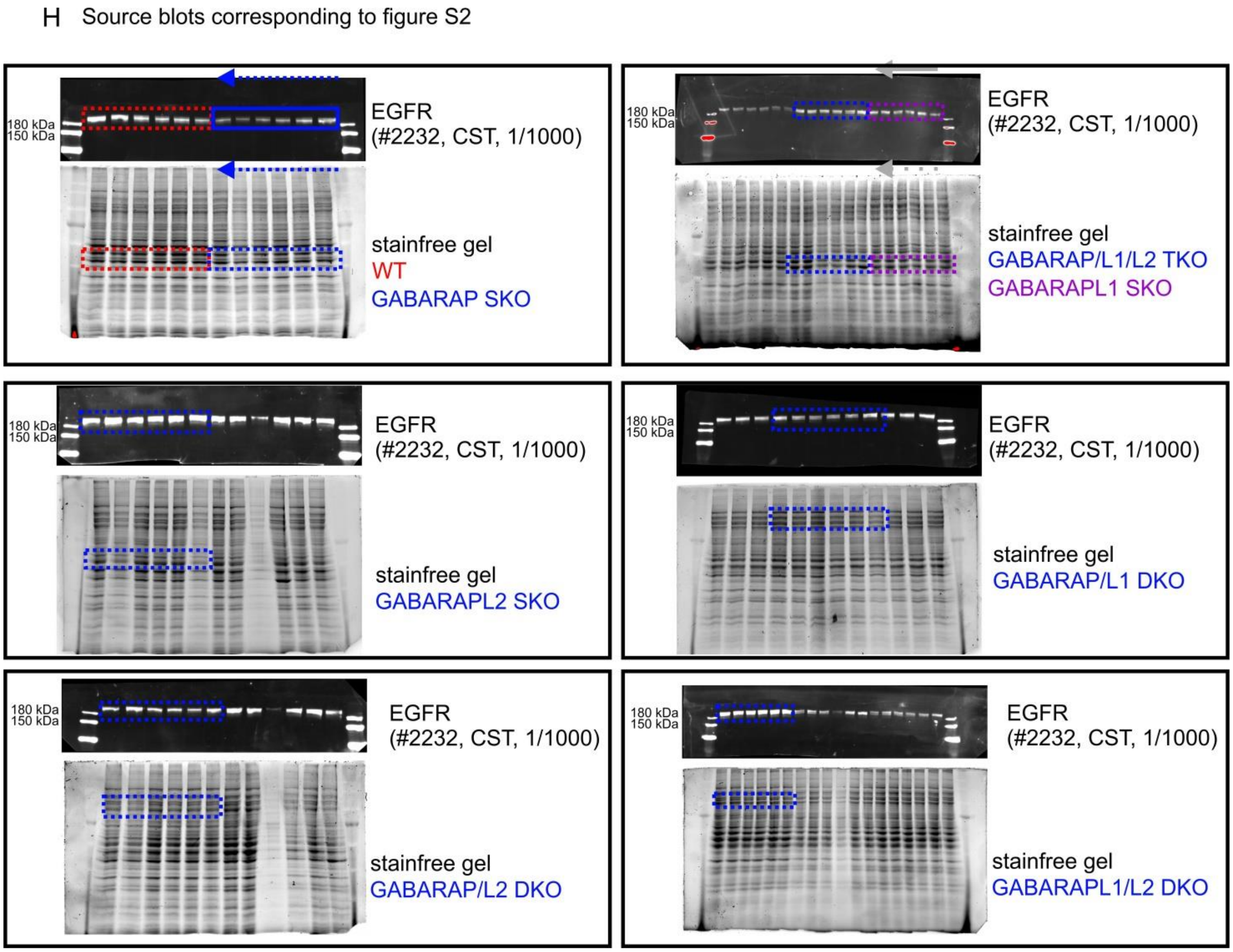

Figure S6: Uncropped source blots of immunoblotting experiments.

This figure contains the source blots corresponding to respective immunoblotting experiments as indicated. (A) Uncropped source blots corresponding to figure 1. (B) Uncropped source blots corresponding to figure 2. (C) Uncropped source blots corresponding to figure 3. (D) Uncropped source blots corresponding to figure 4. (E) Uncropped source blots corresponding to figure 6. (F) Uncropped source blots corresponding to figure 8. (G) Uncropped source blots corresponding to figure S1. (H) Uncropped source blots corresponding to figure S2. Used antibodies are indicated for each blot.
